# Clinical characteristics and prognoses of patients treated surgically for metastatic lung tumors

**DOI:** 10.18632/oncotarget.14822

**Published:** 2017-01-26

**Authors:** Xiaoliang Zhao, Xiaohua Wen, Wei Wei, Yulong Chen, Jianquan Zhu, Changli Wang

**Affiliations:** ^1^ Department of Lung Cancer Tianjin Medical University Cancer Institute and Hospital, Tianjin Lung Cancer Center, Tianjin Key Laboratory of Cancer Prevention and Therapy, National Clinical Research Center for Cancer Tianjin, Tianjin, P.R. China; ^2^ Tianjin University of Traditional Chinese Medicine, Tianjin, P.R. China

**Keywords:** metastatic lung tumor, surgical treatment, prognostic factor

## Abstract

The clinical characteristics of metastatic lung tumors are not well understood. To explore the surgical indications, surgical modes, and factors that influence postoperative outcomes, we analyzed clinical data from 42 patients with metastatic lung tumors who received surgical treatment at Tianjin Medical University Cancer Institute and Hospital between January 2000 and January 2014. Gender, age, nature of resections, surgical mode, smoking index, disease-free intervals (DFIs), number of metastatic lesions, and lymph node metastases were analyzed. Patients were followed for 6 to 98 months. We found that surgical treatment is feasible for resectable metastatic lung tumors, though postoperative radiochemotherapy had no significant effect on postoperative survival rates among patients with metastatic lung tumors. No patients died perioperatively. The 1-year, 3-year, and 5-year survival rates after surgical resection of metastatic lung tumors were 88.1%, 45.7%, and 34.6%, respectively. Univariate analysis indicated that DFIs and lymph node metastasis correlated with patient prognoses, while multivariate analysis indicated these two variables were independent prognostic factors. Thus surgical treatment may be indicated, depending on patients’ specific condition, to lengthen DFIs in patients with metastatic lung tumors with or without evident lymph node metastasis.

## INTRODUCTION

The lung is one of the most common sites for metastasis from malignant tumors elsewhere in the body. Previous reports demonstrated that 25% to 30% of patients with malignant tumors will eventually have pulmonary metastases [1, 2]. Although metastatic lung tumors have certain similarities with some primary lung tumors, the treatment and prognoses of metastatic tumors are totally different from those of primary tumors. Therefore, the evaluation of the clinical pathologic features and prognoses is very important for the treatment of metastatic lung tumors. Few studies have comprehensively analyzed the clinical characteristics and prognoses of metastatic lung tumors.

Surgical resection of pulmonary metastases is a routine treatment, and its use as a part of individual treatment plans for patients with various advanced malignant cancers is increasing [2–4]. For patients with metastatic lung tumors, surgical treatment is a valuable palliative therapeutic option, which in some patients has resulted in postoperative therapeutic effects comparable to those for primary lung cancer. However, the varying clinical characteristics and postoperative therapeutic effects are not well understood.

To explore the factors that cause postoperative therapeutic effects, complete data from 42 patients admitted to our hospital between January 2000 and January 2014 for surgical treatment for metastatic lung tumors were analyzed by use of univariate and multivariate statistical analyses. We found that surgical treatment is feasible for patients with resectable metastatic lung tumors, and postoperative radio/chemotherapy has no significant effect on postoperative survival rates among patients with metastatic lung tumors. Our results also indicated that disease-free intervals (DFIs) and lymph node metastases are independent prognostic factors for patients with metastatic lung tumors, thus suggesting that active surgical treatment should be applied to patients with longer DFIs and no evidence lymph node metastases.

## RESULTS

### Clinicopathological characteristics of patients with metastatic lung tumors

Clinicopathological characteristics of the patients with metastatic lung tumors are shown in Table 1. Of the 42 patients, 25 were males and 17 were females. Ages were 14 to 78 years, with a mean age of 51 years. Before surgery, most (34) of the patients had no marked clinical symptoms, and the remainder (8) of the patients had nonspecific symptoms, including cough, expectoration, and chest distress. Most of the patients were diagnosed with metastatic lung tumors from regular chest X-rays or CT follow-up after treatment for primary tumors in other organs. Of the 42 patients, 35 received thoracotomy and 7 received thoracoscopic surgery (17 received simple metastasectomy and 25 received lobectomy).

**Table 1:** Characteristics of patients who underwent surgery for first pulmonary metastases from extrapulmonary regions

Characteristic	Number	Percentage
**Gender**		
Male	25	59.5%
Female	17	40.5%
**Primary tumor location**		
Breast	8	19.0%
Lung	7	16.7%
Bone	5	11.9%
Esophagus	4	9.5%
Colon and Rectum	4	9.5%
Kidney	3	7.1%
Cervix	3	7.1%
Laryngeal	2	4.8%
Gingiva	2	4.8%
Soft tissue	1	2.4%
Nasopharynx	1	2.4%
Liver	1	2.4%
Skin	1	2.4%
**Surgery type**		
Thoracotomy	17	40.5%
VATS	25	59.5%
**Surgery range**		
Wedge or segmental	17	40.5%
Lobectomy	25	59.5%
**Metastatic site**		
Unilateral	37	88.1%
Bilateral	5	11.9%

Median age: 51; Range:14-78

Clinical records from all the patients included the types of malignant tumors in other organs, treatment history, and pathological diagnoses. All 42 primary tumors were originally from 5 patients with sarcomas, 8 patients with breast cancer, 7 patients with lung cancer, 4 patients with colorectal cancer, 4 patients with esophageal carcinomas, 3 patients with renal cancer, 3 patients with cervical carcinomas, 2 patients with laryngeal carcinomas, and 1 patient each with carcinoma of the parotid gland, nasopharyngeal carcinoma, adamantinoblastoma, soft-tissue squamous cell carcinoma, and hepatoblastoma. Among these patients, 37 had unilateral lung metastases, and 5 had bilateral lung metastases, including 3 patients who had lung metastases synchronously with the primary lesions. Among these 42 patients, 71% (30) had solitary metastases, and 29% (12) had more than one metastasis (Table 2).

**Table 2:** Multivariate analysis for prognostic factors in the patients who underwent surgery for first pulmonary metastases

Prognostic factor		N	5-year survival rate	χ^2^ value	*P* value
**Gender**	Male	25	39.1%	0.336	0.562
	Female	17	32.8%		
**Age**	≤60	34	33.3%	0.055	0.814
	>60	8	39.6%		
**Cigarette index**	≤400	27	36.2%	0.002	0.965
	>400	15	32.8%		
**DFI**	≤24	23	27.3%	5.556	0.018
	>24	19	46.4%		
**Metastatic site**	Unilateral	37	39.2%	2.244	0.134
	Bilateral	5	30.2%		
**Number of metastasis**	Solitary	30	38.7%	1.764	0.184
	Multiple	12	32.1%		
**Surgery type**	Lobectomy	25	35.2%	0.008	0.927
	Local	17	32.4%		
**Lymph node dissection status**	Yes	30	39.6%	1.186	0.276
	No	12	17.9%		
**Lymph node metastasis**	No	20	46.9%	4.719	0.030
	Yes	10	25.0%		
**Postoperative treatment**	No	25	31.4%	0.139	0.079
	Yes	17	36.6%		

### The relation between clinicopathological features and prognosis in patients with metastatic lung tumors

**Figure 1:**
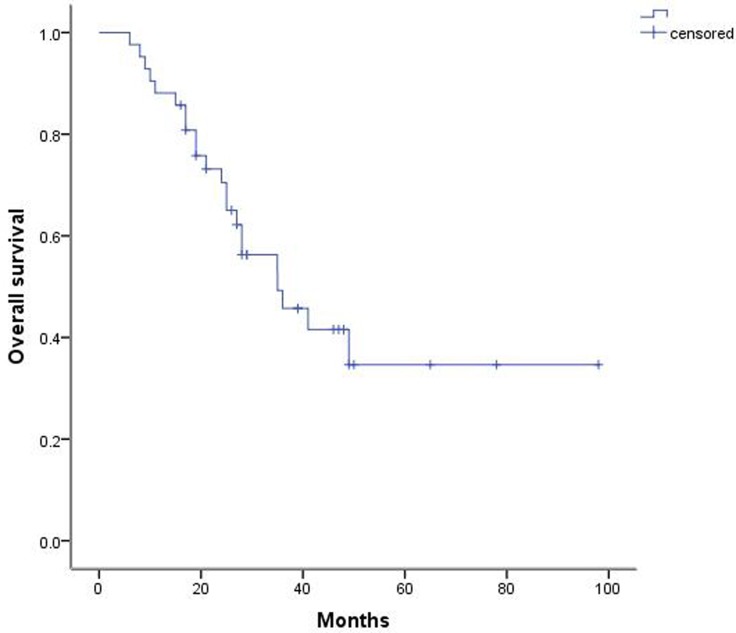
Postoperative survival curve for patients with metastatic lung tumors

**Figure 2:**
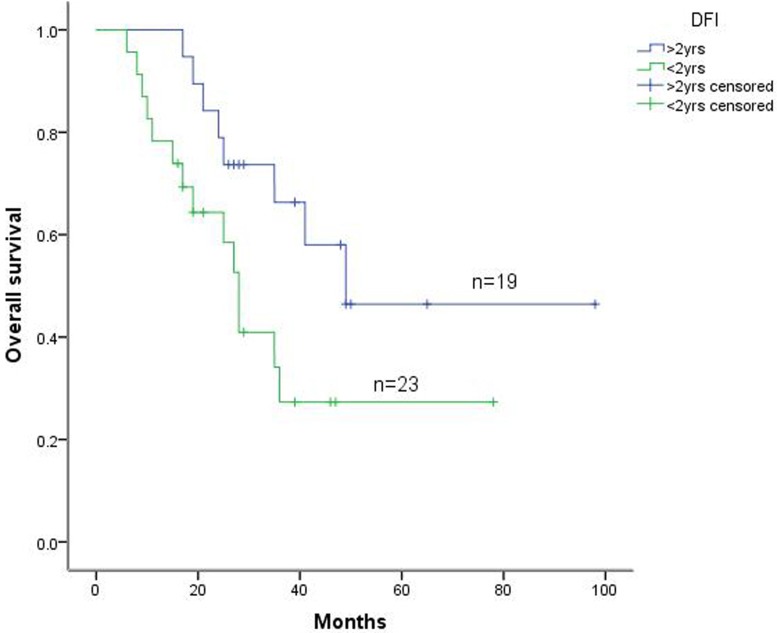
Kaplan-Meier survival curve of patients with lung metastatic tumors according to DFI

Among the 42 patients, 6 experienced postoperative complications, including 3 patients with arrhythmia and 3 patients with pulmonary infection. All patients recovered with active treatment. All 42 patients were then followed for 6 to 98 months. Our results indicated that the 1-year, 3-year, and 5-year survival rates were 88.1%, 45.7%, and 34.6%, respectively, and the median survival was 35 months (Figure 1). In addition, the 5-year survival rate among these patients with DFIs longer than 24 months was 46.4% (19), whereas the 5-year survival rate in patients receiving incomplete resection was 27.3% (23) (Figure 2).

Among these 42 patients, 30 received conventional hilar and mediastinal lymph node dissection, and the other 12 patients did not receive lymph node dissections. After surgery, 10 patients were pathologically observed with lymph node metastases, whereas another 20 patients were observed with no lymph node metastases.

**Figure 3:**
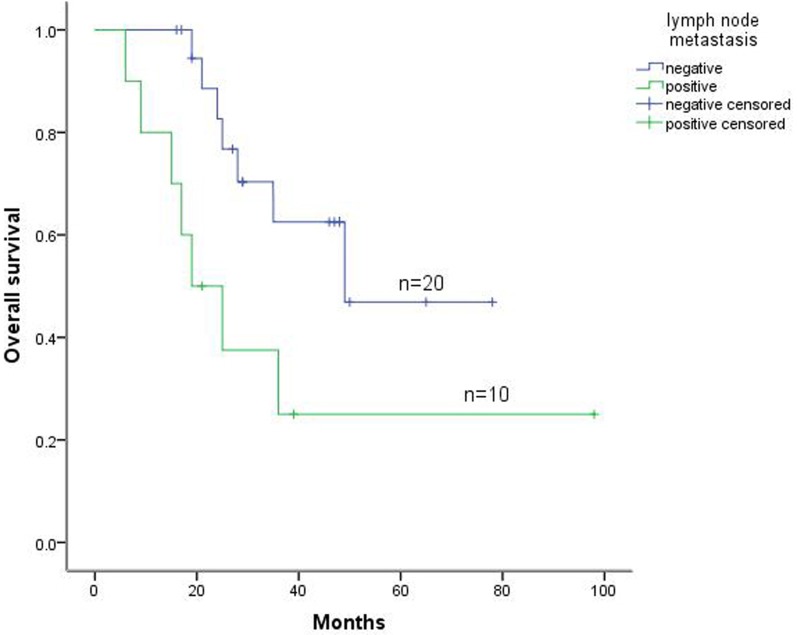
Kaplan-Meier survival curve for lymph node metastasis status of patients with lung metastatic tumors

The 5-year survival rates in patients with and without lymph node metastases were 25.0% (10/30) and 46.9% (20/30), respectively. In addition, the 5-year survival rates in patients with lung metastases of carcinoma and with lung metastases of sarcoma were 37.1% and 24.4%, respectively (Figure 3). Furthermore, Cox multivariate regression analyses of survival revealed DFI and lymph node metastasis as the independent prognostic factors for postoperative survival in patients with metastatic lung tumors (Table 3).

**Table 3:** Cox multivariate regression analysis of survival

	B value	SE	*P* value	Exp (B)	95%CI
DFI	−1.567	0.692	0.017	0.209	0.058–0.714
Lymph node metastasis	1.104	0.537	0.042	3.015	1.052–8.641

## DISCUSSION

Lung metastases often occur during the progression of various malignant tumors, and 25% to 30% of patients with malignant tumors develop lung metastases [1, 2]. With better understanding of metastatic lung tumors and the improved therapeutic approaches for them, favorable therapeutic results have been achieved with active surgical treatment for patients with lung metastases after clinical recovery from the primary tumors [4]. Indeed, previous studies indicated that the 3-year and 5-year survival rates, as well as the therapeutic effects after lung metastasectomy, were much better than those for the primary tumors [4, 5].

Similarly, among patients with metastatic lung tumors who received surgical treatment in the present study, the 3-year and 5-year overall survival rates were 45.7% and 34.6%, respectively, which were much higher than the postoperative survival rates for stage IIIA non-small cell lung cancer (NSCLC). Usually, the 3-year overall survival rate for stage IIIA NSCLC is approximately 24.9% to 33% [6–8]. Therefore, for patients with metastatic lung tumors whose primary tumors have been clinically cured, particularly those with unilateral lung metastases, active surgical treatment might be desirable if the patients’ conditions permit.

Surgical resection, developed as a standard procedure for the treatment of metastatic lung tumors, can prevent further spread and metastases of tumors [2–4]. Although accumulating evidence showing that postoperative factors for metastatic lung tumors, including pathological types of metastatic tumors, DFIs, number of metastatic lesions, tumor doubling time (TDT), and mediastinal lymph node metastases, might be associated with the prognosis of metastatic lung tumors [9, 10], comprehensive surgical indications and their therapeutic effects (for the prognosis of the patients with metastatic lung tumors) are still not well understood. We therefore assumed that the influence of multiple factors, including DFIs or lymph node metastases, might act as independent postoperative prognostic factors for patients with metastatic lung tumors in this process.

From use of either univariate or multivariate analyses (Figures 2 and 3), and generally consistent with previous reports [9, 10], DFIs were reported as important prognostic factors, based on the assumption that DFIs could be correlated with the intensity of the oncogenic factors and the TDTs. The DFI is therefore regarded as an indirect indicator of tumor growth, and longer DFIs usually indicate longer TDTs, which subsequently result in slower tumor progression and longer patient survival [11]. Therefore, better surgical effects for metastatic lung tumors might be achieved for intrapulmonary metastases occurring after effective control of the primary tumors.

In the present study, 30 patients who received lymph-node dissections or sampling were divided into the lymph node-negative group, which included 20 patients, or the lymph node-positive group, which included 10 patients, for statistical analyses. Both univariate and Cox multivariate analyses revealed lymph-node metastases as important prognostic factors (*P* < 0.05), with the 5-year survival rates 46.9% and 25.0%, respectively (Figure 3 and Table 3). Previous studies also indicated significant difference between the lymph-node dissection negative group and positive group in 3-year survival rates [12–14]. However, some retrospective analysis results indicated that lymph-node metastases were significant predictors of poor prognosis, but the extent of lymph-node dissection had no relation to survival [15]. Therefore, systematic mediastinal lymph-node dissections should be performed for prognostic purposes during pulmonary metastasectomies [16]. Our data show that lymph-node metastasis was a negative predictor for 5-year overall survival.

We expected that cancer metastases may occur through either the blood route or the lymph node route, and simple lung metastasectomies could not block the lymph node metastatic route from the primary tumors. Therefore, better prognoses may be achieved in patients undergoing lymph-node dissections while receiving resections of metastatic lung tumors compared with those who did not undergo hilar or mediastinal lymph-node dissections. All this evidence suggests that lymph-node dissection should be mandatory for patients with hilar or mediastinal metastases

Our results are not consistent with previous reports that assume postoperative auxiliary therapy can improve the prognoses of patients with metastatic lung tumors [17]. Our results, showing 5-year overall survival rates of 31.4% and 36.6% without and with postoperative treatment, respectively, indicate only a weak relation between postoperative chemotherapy or radiotherapy and overall survival.

We expected that the conflicting conclusions might be because most patients who received postoperative auxiliary radio/chemotherapy had diseases sensitive to radio/chemotherapy, such as colon carcinomas and breast cancer, whereas those who did not receive radio/chemotherapy had sarcomas or other special types of cancer. In addition, patients who experience substantial variability in biological behavior because of different tumor types, as well as receive postoperative auxiliary therapy, may have varied survival rates compared with patients with metastatic lung tumors.

Several studies indicate that surgical treatment might have fewer side effects for patients with multiple or bilateral lung metastases, thus suggesting that survival rates are significantly better in patients with solitary metastases than in those with multiple metastases, and an increasing number of metastatic lesions might be directly associated with decreased postoperative survival [18, 19]. In contrast, other investigations demonstrated the comparable effects of surgical treatment for patients with multiple or bilateral metastatic lung tumors and those with solitary lung metastases [1, 20].

Our results also indicated no significant difference in the effects of surgical resection between the 12 patients with unilateral or bilateral multiple metastatic lung tumors and the 30 patients with solitary unilateral metastatic lung tumors. By following the patients who died after surgery from multiple lung metastases, we noticed that most of these patients also had metastases to other organs, including bone, liver, and brain. Therefore, for patients with unilateral or bilateral multiple intrapulmonary metastatic tumors, a thorough understanding of the surgical indications is required, and more thorough and accurate examinations of other organs are essential before surgery.

Surgical treatment whenever possible for unilateral or bilateral multiple lung metastases might be reasonable, if extrapulmonary metastases are excluded. Furthermore, Patel et al [21] and McCormack and Ginsberg [22] described how among 448 patients receiving surgical treatment for metastatic lung tumors, 663 thoractomies were performed, including in 2 patients who received as many as 10 thoracotomies, thus suggesting that repeated thoracotomic resections should be allowed for patients with recurrent metastatic lung tumors.

With the progression of combined treatment for tumors, surgical treatment may provide a debulking operation or auxiliary therapy for metastatic lung tumors, thus further expanding the indications for surgical treatment for metastatic lung tumors.

## MATERIALS AND METHODS

### Patients

The complete clinical records of 42 metastatic lung tumor patients who received surgical treatment from January 2000 to January 2014 in Tianjin Medical University Cancer Institute and Hospital (Tianjin, China) were selected for use in this study.

### Follow-up care

None of the 42 patients died perioperatively. After surgery, all patients were strictly followed and given systemic chemotherapy, radiotherapy, or biotherapy, depending on their pathological types. Postoperative survival (in months) was recorded normatively, and any patients lost to follow-up were calculated as deaths. All the patients were followed for 6 to 98 months.

### Statistical analysis

All data were processed by SPSS v19.0 software (SPSS Inc., Chicago, IL). The endpoint evaluated for all the patients was patient survival from the date of operation. Data from patients lost to follow-up were censored at the date of the last observation. The overall survival rate was calculated by the Kaplan-Meier method, and univariate and multivariate analyses were performed for the relevant prognostic and survival factors. The survival curves were estimated by the Kaplan-Meier method and compared statistically by the log-rank test. The Cox proportional hazards model was used for multivariate analysis to investigate the effects of these factors on postoperative survival.
