# Correction: Clinical characteristics and prognoses of patients treated surgically for metastatic lung tumors

**DOI:** 10.18632/oncotarget.26575

**Published:** 2019-01-04

**Authors:** Xiaoliang Zhao, Xiaohua Wen, Wei Wei, Yulong Chen, Jianquan Zhu, Changli Wang

**Affiliations:** ^1^ Department of Lung Cancer Tianjin Medical University Cancer Institute and Hospital, Tianjin Lung Cancer Center, Tianjin Key Laboratory of Cancer Prevention and Therapy, National Clinical Research Center for Cancer Tianjin, Tianjin, P.R. China; ^2^ Tianjin University of Traditional Chinese Medicine, Tianjin, P.R. China

**This article has been corrected:** The correct author contribution information is given below:

Xiaoliang Zhao^1,*^, Xiaohua Wen^2,*^, Wei Wei^1^, Yulong Chen^1^, Jianquan Zhu^1^ and Changli Wang^1^

^1^ Department of Lung Cancer Tianjin Medical University Cancer Institute and Hospital, Tianjin Lung Cancer Center, Tianjin Key Laboratory of Cancer Prevention and Therapy, National Clinical Research Center for Cancer Tianjin, Tianjin, P.R. China

^2^ Tianjin University of Traditional Chinese Medicine, Tianjin, P.R. China

^*^ Xiaoliang Zhao and Xiaohua Wen contributed equally to this work

Original article: Oncotarget. 2017; 8:46491-46497. https://doi.org/10.18632/oncotarget.14822

